# Next-Generation Sequence Assembly: Four Stages of Data Processing and Computational Challenges

**DOI:** 10.1371/journal.pcbi.1003345

**Published:** 2013-12-12

**Authors:** Sara El-Metwally, Taher Hamza, Magdi Zakaria, Mohamed Helmy

**Affiliations:** 1 Computer Science Department, Faculty of Computers and Information, Mansoura University, Mansoura, Egypt; 2 Botany Department, Faculty of Agriculture, Al-Azhar University, Cairo, Egypt; 3 Biotechnology Department, Faculty of Agriculture, Al-Azhar University, Cairo, Egypt; Accelrys, United States of America

## Abstract

Decoding DNA symbols using next-generation sequencers was a major breakthrough in genomic research. Despite the many advantages of next-generation sequencers, e.g., the high-throughput sequencing rate and relatively low cost of sequencing, the assembly of the reads produced by these sequencers still remains a major challenge. In this review, we address the basic framework of next-generation genome sequence assemblers, which comprises four basic stages: preprocessing filtering, a graph construction process, a graph simplification process, and postprocessing filtering. Here we discuss them as a framework of four stages for data analysis and processing and survey variety of techniques, algorithms, and software tools used during each stage. We also discuss the challenges that face current assemblers in the next-generation environment to determine the current state-of-the-art. We recommend a layered architecture approach for constructing a general assembler that can handle the sequences generated by different sequencing platforms.

## Introduction

The field of biological research has changed rapidly since the advent of massively parallel sequencing technologies, collectively known as next-generation sequencing (NGS). These sequencers produce high-throughput reads of short lengths at a moderate cost [1], [2] and are accelerating biological research in many areas such as genomics, transcriptomics, metagenomics, proteogenomics, gene expression analysis, noncoding RNA discovery, SNP detection, and the identification of protein binding sites [3]–[5].

The genome assembly problem arises because it is impossible to sequence a whole genome directly in one read using current sequencing technologies. The shotgun sequencing method breaks a whole genome into random reads and sequences each read independently. The process of reconstructing a whole genome by joining these reads together up to the chromosomal level is known as genome assembly. For almost 30 years, the Sanger method was the leading technology in genome sequencing. This method generates low-throughput long reads (800–1000 bp) with high costs [1], [6]. Since the emergence of next-generation sequencing technology, sequencers can produce vast volumes of data (up to gigabases) during a single run with low costs. However, most of the produced data is distorted by high frequencies of sequencing errors and genomic repeats. Thus, building a genome assembler for a next-generation environment is the most challenging problem facing this technology due to the limitations of the available computational resources for overcoming these issues. The first step toward overcoming the assembly challenge of NGS is to develop a clear framework that organizes the process of building an assembler as a pipeline with interleaved stages. The NGS assembly process comprises four stages: preprocessing filtering, a graph construction process, a graph simplification process, and postprocessing filtering [7]–. A series of communication messages are transferred between these stages and each stage works on its respective inputs to produce the outputs that reflect its function. These stages are found in most working assemblers (see below) in the next-generation environment but some assemblers delay preprocessing filtering until the later stages. In this review, we discuss the complete framework and address the most basic challenges in each stage. Furthermore, we survey a wide range of software tools, which represent all of the different stages in the assembly process while also representing most of the paradigms available during each stage. Most of the tools reviewed are freely available online as open-source projects for users and developers.

## Next-Generation Sequencing Technologies

The revolution in DNA sequencing technology started with the introduction of second-generation sequencers. These platforms (including 454 from Roche; GA, MiSeq, and HiSeq from Illumina; SOLiD and Ion Torrent from Life Technologies; RS system from Pacific Bioscience; and Heliscope from Helicos Biosciences) have common attributes such as parallel sequencing processes that increase the amount of data produced in a single run (high-throughput data) [5], . They also generate short reads (typically 75 bp for SOLiD [37], 100 to 150 bp for Illumina [38], ∼200 bp for Ion Torrent [38], and 400 to 600 bp for 454 [38]) and long reads of up to 20 kb (with Pacific Bioscience) but with higher error rates [1], [16], . Thus, each platform also has a common error model for the data they generate, such as indels for 454, Ion Torrent, and Pacific Bioscience platforms and substitutions for SOLiD and Illumina [6], [39]. Each platform generally produces two types of data: 1) the short-read sequences and 2) the quality score values for each base in the read. The quality values are used to assess the sequence quality, trim reads, and remove low-quality bases. Several next-generation platforms can produce paired-end reads, which are libraries that contain the sequences corresponding to both ends of the read. Each paired-end has a separation distance, which is estimated using a library preparation protocol during the sequencing process. This separation distance is known as the insert size or clone length. These paired-end reads are used to combine contigs in the later stages of the genome assembly process and they are employed as a measure for testing the quality of the assembled genome. Next-generation sequence reads are typically available online at the Sequence Read Archive (SRA) [40], while the assembled reads are available at the Assembly Archive [41] and the descriptions of assembled contigs and scaffolds are available in AGP files [42].

## Genome Assembly Pipeline

Treating the genome assembly problem as a jigsaw puzzle provides useful insights into the different challenges encountered during assembly. The first challenge is to place each read (piece) in the correct position in the puzzle, which will affect the quality of puzzle solving because the only available information for determining the correct position of a read (piece) comes from its neighbors. The second challenge is the increased number of reads (pieces) in the puzzle, which will increase the complexity of determining the correct position. The third challenge is the ambiguity that results from positioning similar reads (pieces), which share similar suitable locations in the puzzle. Finally, some reads (pieces) have unique features and they serve as unique indicators to their locations in the puzzle [27].

**Figure 1:**
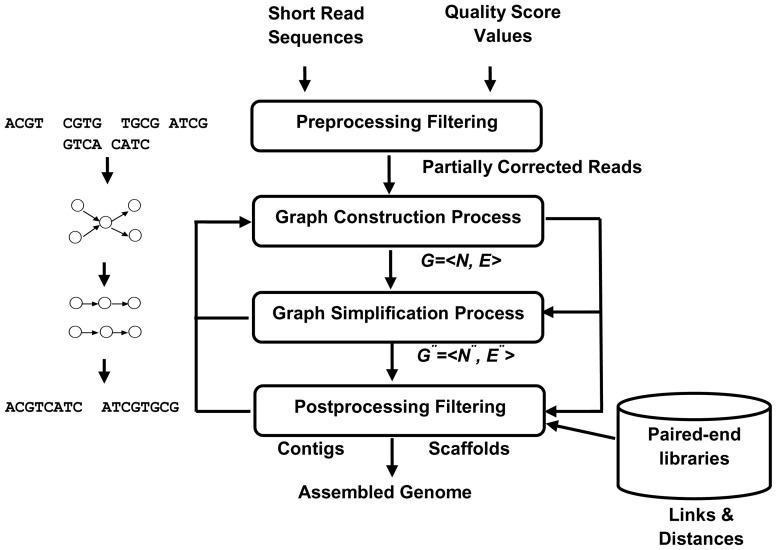
Schematic representation of the four stages of the next-generation genome assembly process. Note: *G″* is a simplified version of graph *G* with *N* nodes and *E* edges.

Next-generation genome assembly begins with a set of short reads, which may contain errors depending on the experimental sequencing procedures. These reads are joined together to form longer contiguous reads known as *contigs* by a computer program known as an *assembler*. These contigs are joined together to form longer contigs known as *scaffolds* (see Figure 1) [22].

There are two approaches for genome assembly: the comparative approach and the *de novo* approach. During comparative assembly, also known as reference-based assembly, a reference genome from the same organism or a closely related species is used as a map to guide the assembly process by aligning the fragments being assembled. This approach is used in resequencing applications, for example [43]. During *de novo* assembly, no map or guidance is available for assembling the genome, so this approach represents assembly in the strict sense. Therefore, *de novo* assembly is used to reconstruct genomes that are not similar to previously sequenced genomes [20].

To build an assembler, we must know the inputs of the assembler, which are generally two files that contain the sequence reads being assembled and their quality scores (or one file that contains both). Next-generation sequencing technologies have high-throughput short reads so dealing with them is a highly memory-intensive task. To simplify the assembly process and also save time and memory costs, most assemblers format their input data using graph data structures. However, different assemblers differ with respect to their initial graph construction, configuration, traversing, and simplification processes [44].

In the present review, genome assembly is discussed as a single coherent framework that combines the four basic stages of next-generation genome assembly: preprocessing filtering, a graph construction process, a graph simplification process, and postprocessing filtering. Preprocessing filtering is responsible for detecting and correcting erroneous reads before the assembly begins. The graph construction process is responsible for creating a graph model, which is used to organize short-read sequences into a compact form and to create longer reads during assembly. The graph simplification process is used to simplify the graph by reducing the number of graph nodes and edges, and removing erroneous ones. Postprocessing filtering builds contigs, detects misassembled ones, and extends them into scaffolds. In this stage, the paired-end reads are incorporated into filter contigs by creating a contig connectivity graph or using a previously constructed one (in the second stage) based on the updated information. The new graph or the previous graph must be filtered one step further after incorporating paired-end constraints that detect misassembled contigs and unresolved repeats, which is indicated by the arrows between the three lower stages in Figure 1.

The current assemblers used in the next-generation environment have some or all of these basic stages. Furthermore, stand-alone preprocessing filters (error correction tools [45]–[51]) and postprocessing filters (scaffolders [52]–[57]) are available, while some assemblers have their own preprocessing and postprocessing modules. Some assemblers delay the error correction step until the graph simplification stage because some errors are not visible until the graph has been started, e.g., to distinguish polymorphisms from sequencing errors. Furthermore, performing parallel error correction operations during the graph construction process for a whole set of reads will reduce the overall computational time [14], [35]. Some assemblers rely on correcting the errors early, which may simplify the graph construction process and reduce the graph size. Some errors are also not detected during the graph simplification step so detecting them early helps to remove them from the read sets before the graph creation stage. During postprocessing filtering, some assemblers use stand-alone scaffolders to assemble the contigs one step further into scaffolds whereas other have their own scaffolding modules that produce scaffolds from contigs directly. In many traditional assembly pipelines, error correction or graph simplification phases are absent. The long reads of the first-generation sequencers, compared with most of the next-generation sequencers, contribute positively to the absence of these phases. With long reads, assemblers can detect long overlaps, which limits the influence of sequencing errors even if the overlap sequences are inexact. In addition, using a set of assembly parameters for validating the overlaps among long reads in the global alignment process is sufficient to detect these sequencing errors or simply ignore them. If those errors are ignored, the computation of contigs consensus sequences promises their detection, by mapping reads back to contigs. Moreover, these isolated errors do not affect the topology of the created assembly graph [43], [58].

## Preprocessing Filtering

**Figure 2:**
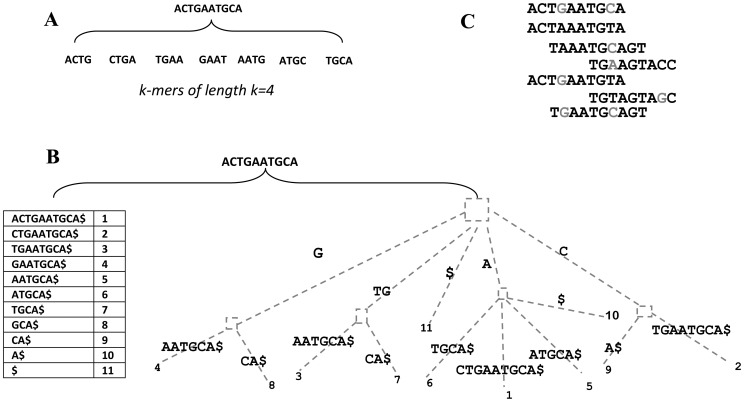
Different approaches for error corrections. (A) K-spectrum approach: a set of substrings of fixed length k are extracted from the read and ready to filter. (B) Suffix tree/array approach: a set of substrings of different lengths of k (suffixes) are extracted from the read, represented in the suffix tree, and ready to filter. (C) Multiple sequence alignment approach: reads are aligned to each other to define consensus bases and correct erroneous ones.

The goal of the preprocessing filter is to correct or eliminate erroneous reads before starting the assembly process. These errors are caused by the sequencing platforms and, therefore, they vary among platforms. The different errors targeted by preprocessing filters include substitutions (mismatch), indels (insertion/deletion), and ambiguous bases (i.e., N). Detecting and correcting these errors early will facilitate the assembly process and prevent misassembled contigs in the later stages. Error correction algorithms vary from simple trimming processes using base quality scores to complex error correction approaches based on the frequency of erroneous reads in the set being assembled [39]. All error correction algorithms are based on the same general concept that reads with errors are infrequent and random so they can be detected by counting the reads in the assembly pool. Low-frequency reads are candidates for error correction algorithms and are aligned to high-frequency reads that share substrings. However, this idea is affected by the challenges of high-frequency genomic repeats and nonuniform sampling of the genome, which lead to ambiguous results derived from multiple equal correction choices. There are four basic approaches to error correction: the K-spectrum approach, Suffix Tree/Array approach, Multiple Sequence Alignment approach, and Hybrid approach (see Figure 2) [45]–[51]. These error correction approaches and their implemented tools (see Table 1) are discussed in detail in the following sections.

**Table 1:** Preprocessing filters: Practical and technical comparisons.

Preprocessing Filters	Operating System	Programming Language	Single PC/Cluster	Open-Source	Targeted Errors	Sequencing Platform	Input File Formats	Output File Formats	Websites	Ref.
Quake* **(V0.3)**	Linux (64 bits)	C++, Python	Single	Y	Substitution	Illumina	fastq	fastq	http://www.cbcb.umd.edu/software/quake	[47]
Reptile* **(V1.1)**	Linux (64 bits)	C++, Perl	Single	Y	Substitution	Illumina	fastq	.fa, .errors**	http://aluru-sun.ece.iastate.edu/doku.php?id=reptile	[51]
Hammer* **(V0.2)**	Linux (64 bits)	C++, Perl	Single	Y	Substitution	Illumina	fastq	raw k-mers	http://bix.ucsd.edu/projects/hammer	[48]
SHREC **(V2.2)**	Linux (64 bits)/Mac OS X/Windows	Java	Single/Cluster	Y	Substitution	Illumina	fastq [S]	fastq [S]	http://sourceforge.net/projects/shrec-ec	[50]
HiTEC* **(V1.0.2)**	Linux (64 bits)	C++	Single	Y	Substitution	Illumina	fasta fastq	fasta	http://www.csd.uwo.ca/~ilie/HiTEC	[45]
Coral* **(V1.4)**	Linux	C++	Single	Y	SubstitutionInsertionDeletion	Any platform	fasta fastq	fasta/fastq	http://www.cs.helsinki.fi/u/lmsalmel/coral	[49]
ECHO*	Linux/Mac OS X/Windows	C++, Python	Single	Y	Substitution	Illumina	.txt	fastq	http://uc-echo.sourceforge.net	[46]
Hybrid-SHREC	Linux (64 bits)	Java	Single	Y	SubstitutionInsertionDeletion	Any platform	fasta***	fasta	http://www.cs.helsinki.fi/u/lmsalmel/hybrid-shrec/	[62]
PBcR*	Linux (64 bits)	C, C++, Perl	Single/Cluster	Y	SubstitutionInsertionDeletion	PacBio RS454Illumina	fastq****SFFfasta	FRGfastaqualfastq	http://sourceforge.net/apps/mediawiki/wgs-assembler/	[16]

* Personal communications with authors.

** Files recording error positions and bases can be converted into fasta/fastq.

*** Supported base or color space.

**** Illumina and PacBio RS formats; also, there are tools for converting fasta files to fastq-compatible files.

[S] Speculated, based on sequencing platforms.

### K-Spectrum Approach

K-spectrum–based filters extract the set of all k-mers (substring of length k) from the reads, which is known as the k-spectrum (see Figure 2A) [47], [48], [51]. The k-mers with small differences (hamming distances) among them are probably from the same genomic position so they are candidates for correcting errors depending on their frequencies. K-spectrum–based filtering starts by extracting all of the k-mers from the set of reads being assembled. A weight value is assigned to each k-mer depending on several factors, such as its frequency and the quality scores of the bases in the k-mers. Subsequently, the k-mers are sorted according to their weights and a suitable threshold (cutoff point) is determined that separates trusted and untrusted k-mers. The reads that contain untrusted k-mers are treated as candidates for the error correction filter. The filter tries to convert untrusted k-mers into trusted ones using a minimum number of editing operations. The conversion process is repeated until there are no more untrusted k-mers. Thus, all of the retained reads contain trusted (error-free) k-mers.

The general k-spectrum–based approach has been implemented in many stand-alone software packages for error corrections such as Quake [47], Reptile [51], and Hammer [48] but with differences in their implementations. Also, the same approach has been implemented as a built-in component for error correction in short-read assemblers such as Euler-SR [8]–[10], [26], [27], ALLPATHS-LG [7], [18], [59], SOAPdenovo [17], SGA [30], Readjoiner [13], and Fermi [60]. Table 1 and Table 2 list several technical and practical features of stand-alone and built-in error correction tools, respectively.

**Table 2:** Next-generation genome assemblers: Architecture.

Assemblers	Preprocessing Filtering	Graph Construction Process	Graph Simplification Process	Postprocessing Filtering	Ref.
**Newbler** ***GS de novo assembler***	N/A	Overlap-based	-Merging consecutive nodes	-Building contigs	[19]
**Edena**	N/A	Overlap-based	-Removing dead ends-Removing transitive edges-Dealing with bubbles	-Building contigs	[14]
**Celera** **CABOG** ***wgs-assembler***	-Remove and correct erroneous reads	Overlap-based	-Merging consecutive nodes-Removing dead ends	-Building contigs-Detecting misassembled contigs-Merging contigs and fill gaps-Removing transitive edges-Detecting repeated contigs-Building scaffolds	[21], [23]
**Shorty**	N/A	Overlap-based	N/A	-Building contigs-Detecting misassembled contigs-Merging contigs and fill gaps-Building scaffolds	[15]
**Forge**	-Remove erroneous reads	Overlap-based	N/A	-Building contigs-Building scaffolds	[11]
**SGA**	-Remove and correct erroneous reads	Overlap-based	-Removing dead ends-Dealing with bubbles	-Building contigs-Building scaffolds	[30]
**Readjoiner**	-Remove and correct erroneous reads	Overlap-based	-Removing dead ends-Dealing with bubbles	-Building contigs	[13]
**Fermi**	-Correct erroneous reads	Overlap-based	-Dealing with bubbles	-Building contigs	[60]
**Euler-SR**	-Remove and correct erroneous reads	K-mer–based	-Merging consecutive nodes-Removing dead ends-Dealing with bubbles-Removing tangles	-Building contigs-Building scaffolds	[10]
**ALLPATHS-LG**	-Remove and correct erroneous reads	K-mer–based	-Removing dead ends-Dealing with bubbles	-Building contigs-Building scaffolds	[7], [18], [59]
**Velvet**	-Remove erroneous reads	K-mer–based	-Merging consecutive nodes-Removing dead ends-Dealing with bubbles	-Building contigs-Merging contigs and fill gaps-Detecting and resolving repeated contigs-Building scaffolds	[35]
**ABySS**	N/A	K-mer–based	-Removing dead ends-Dealing with bubbles	-Building contigs-Merging contigs	[31]
**SOAPdenovo**	-Correct erroneous reads	K-mer–based	-Merging consecutive nodes-Removing dead ends-Dealing with bubbles-Removing tangles	-Building contigs-Merging contigs and fill gaps-Removing transitive edges-Detecting repeated contigs-Building scaffolds	[17]
**SparseAssembler**	N/A	Sparse k-mer–based	-Removing dead ends-Dealing with bubbles	-Building contigs	[34]
**SSAKE**	N/A	Greedy-based	N/A	-Building contigs	[33]
**SHARCGS**	-Remove erroneous reads	Greedy-based	N/A	-Building contigs	[12]
**Vcake**	N/A	Greedy-based	N/A	-Building contigs	[74]
**QSRA**	N/A	Greedy-based	N/A	-Building contigs	[75]
**Taipan**	N/A	Hybrid-based	-Removing transitive edges	-Building contigs	[29]

### Suffix Tree/Array Approach

Suffix tree/array–based filters generalize the k-mer idea by using different values of k, which represent different suffixes (substrings) in the reads [45], [50]. Rather than storing/retrieving fixed k-mers with their frequencies in a hash table, suffix tree/array–based filters store/retrieve variable-size k-mers with their frequencies in a suffix tree/array. The suffix array is also more space-efficient than the suffix tree. The suffix tree/array filter starts by extracting all suffixes from the reads and computing their frequencies. The suffixes and their frequencies are organized in a tree/array data structure (see Figure 2B). Next, the tree/array is traversed to search for erroneous nodes (suffixes) with frequencies less than the specified threshold. The filter tries to find the most similar nodes in the neighbors, which serve as candidate solutions for correction. If there are no candidate solutions for correction, the reads corresponding to erroneous nodes are removed from the read set.

The suffix tree/array approach has been implemented in many stand-alone software packages for error corrections such as SHREC [50] and HiTEC [45] (see Table 1).

### Multiple Sequence Alignment (MSA) Approach

The idea behind this approach is using sequence alignment to detect and correct erroneous reads by aligning them with a reference genome or each other, as explained below (see Figure 2C) [46], [49]. Reads that share substrings (k-mers) are likely to be similar, while those that have high-frequency k-mers are likely to be correct and are used as candidate solutions to correct reads with low-frequency k-mers. The consensus (correct) bases are determined by aligning erroneous reads with the trusted ones, thereby correcting the errors.

The MSA approach has been implemented in many stand-alone software packages for error corrections such as Coral [49] and ECHO [46] (see Table 1). Also, the same approach has been implemented as a built-in component for error correction in short-read assemblers such as CABOG [21] (see Table 2).

### Hybrid Approach

The idea behind this approach is combining the complementary attributes of next-generation sequencing techniques to detect and correct erroneous reads [11], [61]. These attributes include the long reads from the 454 platform and the high indel error rates compared with Illumina reads. These longer reads can be used to detect overlaps during *de novo* assembly. The Illumina reads are shorter but they have high coverage and can be used to detect and correct erroneous reads [6]. Early hybrid techniques were based on combining the reads from first- and second-generation sequencers such as Sanger with 454, or Illumina reads [11]. The continuous improvement of NGS technologies had increased the read lengths and the hybrid techniques among them have been developed such as PBcR [16], which is a hybrid error correction method for erroneous reads from PacBio RS that uses high-quality short reads produced by the same sequencer or other sequencers, such as 454 or Illumina reads (see Table 1). PBcR aligns short reads against the longer ones and searches for a maximum matching between them to create a consensus sequence. This method has been integrated with Celera [23] to assemble different prokaryotic and eukaryotic genomes. Hybrid-SHREC [62] deals with different error models produced by the next-generation sequencers, e.g., substitution for Illumina and SOLiD and indels for 454 (see Table 1). It relies on aligning these reads together for correcting various models of errors using the suffix array approach.

Recently, Yang *et al.* [39] evaluated various stand-alone error correction methods, representing different approaches, and reported that most of them targeted the substitution errors due to the abundant usage of Illumina sequencing reads (see Table 1); among them Reptile, HiTEC, and ECHO produce the best results. While Coral and Hybrid-SHREC are the only tools targeting indels errors with better results produced from Coral, they still need improvements in their substitution error correction results compared with others.

Another interesting evaluation study [63] shows that some assemblers, such as ABySS, SOAPdenovo, Velvet, and CABOG, produce improved results using a separate program for error correction while others, such as SGA, are most effective with their built-in modules for error correction. Further, the study mentioned that the built-in error correction module in ALLPATHS-LG produce more accurate reads than the stand-alone tool Quake.

It should be noted that there are many challenges facing the current error correction modules such as user-independent parameter selection, distinguishing sequencing errors from polymorphisms, dealing with different data sets with different attributes (read length, error rates and error models, genomic coverage), using of paired-end reads to overcome genomic repeats, and improving the performance of error correction algorithms (the time and memory costs) toward the increasing throughput of the next-generation sequencers [39]. Furthermore, the field of error correction still needs deeper assessment of various stand-alone error correction tools against built-in error correction modules in different assemblers.

## Graph Construction Process

In this stage, the reads are partially corrected and filtered, which makes them suitable for the assembly process. The goal of the assembly process is to combine these partially corrected reads to form longer contiguous reads, which are technically referred to as contigs. The combined reads are those sharing nucleotides at their ends, i.e., merged reads share an overlap region. Most NGS assemblers format their input short reads as graph data structures but they differ in their initial graph construction, configuration, traversing, and simplification processes. The graph is an abstract data structure, which describes the similarity relations within a set of reads. Mathematically, a graph is represented as a set of vertices (nodes) and edges. In the assembly graph, the nodes represent strings or substrings of reads, while the edges represent the suffix-to-prefix overlaps between reads [64], [65]. There are many approaches to graph construction, which can be classified as overlap-based construction, k-mer–based construction, greedy-based construction, and hybrid-based construction. These approaches are also known as overlap graphs, de Bruijn graphs, greedy graphs, and hybrid graphs, respectively. We will discuss the different approaches to graph construction in the following sections.

### A. Overlap-Based Construction

**Figure 3:**
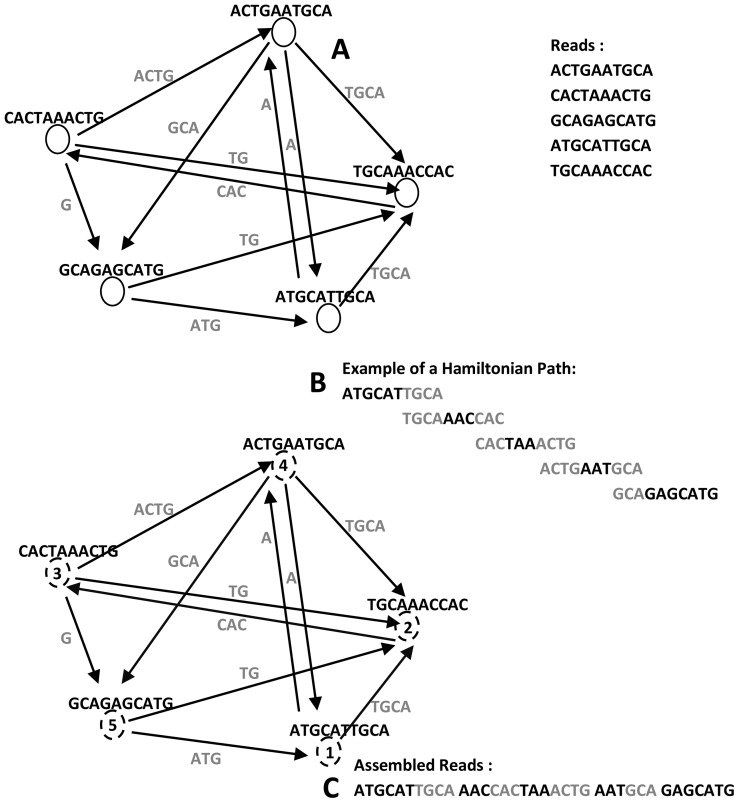
Overlap-based approach for graph construction. (A) Overlap graph where nodes are reads and edges are overlaps between them. (B) Example of a Hamiltonian path that visits each node (dotted circles) exactly once in the graph (note: starting node is chosen randomly). (C) Assembled reads corresponding to nodes that are traversed on the Hamiltonian path.

A classical overlap-based approach for *de novo* assembly consists of three stages: overlap, layout, and consensus (i.e., OLC) [66]. Assemblers following this paradigm start by detecting the overlaps among the set of unassembled reads. Then, the overlap information is organized into a graph where nodes correspond to reads and edges encode the (suffix-to-prefix) overlaps among them. The goal of the layout step is to find a shortest Hamiltonian path that visits each node in the graph exactly once and hence this path represents a solution to the assembly problem. Finally, the overlaps between the reads (nodes) are combined in the consensus step (see Figure 3).

Another alternative representation of a classical overlap graph is a string graph, which is a simplified version constructed from only irreducible (nontransitive) edges [67]. When the transitive edges are reduced, the Hamiltonian path does not represent the solution to the assembly problem. Since there are no assemblers to try to find the optimal path in the assembly graph using a whole set of reads, the solution to the assembly problem is theoretically NP-hard [65].

The minimum overlap length plays a key role in the success of an assembly algorithm. Since the small values will increase the branching nodes in the graph by increasing the frequency of false overlaps, the large values will increase the dead ends by increasing the frequency of nonoverlapped reads [14].

This paradigm is widely used with long reads that have sufficient characters to detect overlaps such as those produced by Sanger and 454 technologies and previously raised concerns about the quadratic complexity of the overlap computation phase [11], [27]. With the advent of a string indexing data structure called FM-index, which can efficiently find overlaps faster than quadratic time, the performance of overlap-based assemblers (e.g., SGA [30] and Readjoiner [13]) has been improved for short-read sequence assembly [68].

This paradigm is implemented in several short-read assemblers such as Newbler [19], CABOG [21], Shorty [15], Forge [11], Edena [14], SGA [30], Fermi [60], and Readjoiner [13]. Table 2, Table 3, and Table 4 list several technical and practical features of these tools.

**Table 3:** Next-generation genome assemblers: Technical comparison.

Assemblers	Operating System	Programming Language	Single PC/Cluster	Open-Source	Website
**Newbler** * **(V2.8)** ***GS de novo assembler***	Linux (32–64) bitsCentOS or RedHat	C++	Single	N	http://454.com/contact-us/software-request.asp
**Edena (V3.121122)**	Linux (32–64) bitsWindows	C++	Single	Y	http://www.genomic.ch/edena
**Celera** * **(V7.0)** **CABOG** ***wgs-assembler***	Linux/Unix (64) bitsMac OS X, Darwin, FreeBSD	C++/C/Perl	Single/Cluster	Y	http://wgs-assembler.sourceforge.net/
**Shorty (V2.0)**	Windows, Linux,Mac OS X**	C++	Single	Y	http://www.cs.sunysb.edu/~skiena/shorty
**Forge** *	Windows, Linux, Mac	C++	Single/Cluster	Y	N/A
**SGA** *	Linux, Mac OS X	C++	Single/Cluster	Y	https://github.com/jts/sga
**Readjoiner** * **(V1.2)**	Linux (32–64), Mac OS X, Cygwin, POSIX-compatible	C	Single/Cluster	Y	http://www.zbh.uni-hamburg.de/readjoiner
**Fermi**	Linux	C	Single	Y	https://github.com/lh3/fermi
**Euler-SR**	Linux (32–64) bits**	C++/Perl**	Single**	N/A**	N/A
**ALLPATHS-LG** *	Linux (64) bits	C++	Single	Y	http://www.broadinstitute.org/software/allpaths-lg/blog
**Velvet** * **(V1.2.08)**	Linux (32–64), Mac OS X, CygwinSparc/Solaris	C	Single	Y	http://www.ebi.ac.uk/~zerbino/velvet
**ABySS (V1.3.4)**	For all platforms	C++	Single/Cluster	Y	http://www.bcgsc.ca/platform/bioinfo/software/abyss
**SOAPdenovo (V1.05)**	Linux (32–64), Mac**	C/C++**	Single	Y	http://soap.genomics.org.cn/soapdenovo.html
**SparseAssembler** *	Linux (64) bits	C/C++	Single	Y	http://sites.google.com/site/sparseassembler/
**SSAKE (V3.8)**	For all platforms	Perl	Single	Y	http://www.bcgsc.ca/bioinfo/software/ssake
**SHARCGS** *	Linux/Unix (32–64)	Perl	Single	Y	http://sharcgs.molgen.mpg.de/
**Vcake** *	Windows (32–64) bitsLinux/Unix (32–64) bits	Perl/C	Single	Y	http://sourceforge.net/projects/vcake/
**QSRA (V1.0)**	Linux/Unix (32–64)	C++	Single	Y	http://mocklerlab.org/tools/2
**Taipan** *	Linux	C	Single	Y	http://taipan.sourceforge.net

* Personal communications with authors.

** Users' experiences and communities' websites.

**Table 4:** Next-generation genome assemblers: Practical comparison.

Assemblers	Sequencing Platform	Input File Format	Output File Format	Genome/Transcriptome	Prokaryotic/Eukaryotic	Single/Paired-End Reads
**Newbler** * ***GS de novo assembler***	Any platform	.sff, .fasta, .qual	.fna, .qual, .txt, .sff, .tsv, .ace	Genome[T]	Prokaryotic	S/P
**Edena**	Illumina/Solexa	.fasta**	.fasta**, .cov, .info	Genome	Prokaryotic	S/P
**Celera** * **CABOG** ***wgs-assembler***	Sanger, Illumina/Solexa, 454, Ion Torrent, Pacific Biosciences	.fasta.fastq.frg, .sff	.asm.fasta .posmap, .qc	Genome	Eukaryotic/Prokaryotic	S/P
**Shorty**	SOLiDIllumina/Solexa, Helicos	.fasta	.fasta	Genome	Prokaryotic	S/P
**Forge** *	Hybrid of Sanger, 454, and Illumina/Solexa	.fasta, .fastq, .qual	.fasta, .txt	Genome	Eukaryotic/Prokaryotic	S/P
**SGA** *	Illumina/Solexa	.fastq	.fasta	Genome	Eukaryotic/Prokaryotic	S/P
**Readjoiner** *	Illumina/Solexa***	.fasta, .fastq	.fasta, .dot, .sga	Genome	Eukaryotic/Prokaryotic	S/P
**Fermi**	Illumina/Solexa	.fastq	.fastq-like format	Genome	Eukaryotic/Prokaryotic	S/P
**Euler-SR**	454Illumina/Solexa	.sff**, .fastq, .eland	.fasta**	Genome	Eukaryotic/Prokaryotic	S/P
**ALLPATHS-LG** *	Illumina/Solexa, Pacific Biosciences	.fastb, .qualb, .pairs	.fasta, .efasta	Genome	Prokaryotic/Eukaryotic	S/P
**Velvet** *	454, Illumina/Solexa, SOLiD	.fasta, .fastq, .fasta.gz, fastq.gz, .sam, .bam, .eland, .gerald	.fasta, .afg, .txt	Genome	Prokaryotic/Eukaryotic	S/P
**ABySS**	Illumina/Solexa454SOLiD	.fastqm, .fasta, .qseq, .export, .sam, .bam	.fasta, .hist, .dot, .adj.dist, .path, coverage.hist	Genome[T]	Eukaryotic/Prokaryotic	S/P
**SOAPdenovo**	Illumina/Solexa	.fastq, .fasta	.contig.scafSeq	Genome[T]	Eukaryotic/Prokaryotic	S/P
**SparseAssembler** *	Illumina/Solexa	.fasta, .fastq	.fasta	Genome	Eukaryotic/Prokaryotic	S
**SSAKE**	Illumina/Solexa	.fasta, raw	.fasta[S]	Genome	Eukaryotic/Prokaryotic	S/P
**SHARCGS** *	Illumina/Solexa	.fasta, raw	.fasta	Genome	Eukaryotic/Prokaryotic	S
**Vcake** *	Illumina/Solexa	.fasta, raw	.fasta	Genome	Prokaryotic	S
**QSRA**	Illumina/Solexa	.fasta, .raw	.fasta[S]	Genome	Eukaryotic/Prokaryotic	S
**Taipan** *	Illumina/Solexa	.raw	.fasta	Genome	Prokaryotic	S

* Personal communications with authors.

** Users' experiences and communities' websites.

*** Available for other sequencing platforms if the datasets are filtered.

[T] Transcriptome assembly version is available.

[S] Speculated, based on sequencing platforms.

### B. K-Spectrum–Based Construction

**Figure 4:**
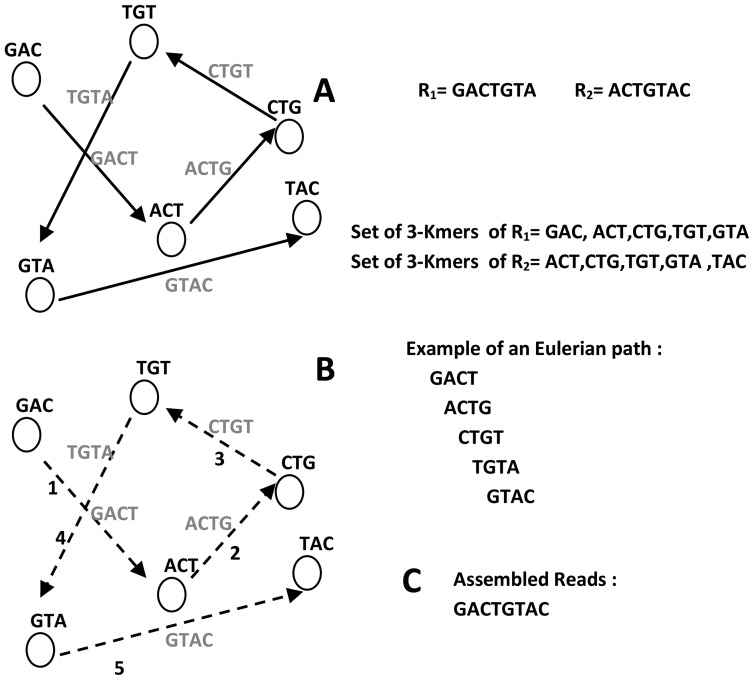
K-spectrum–based approach for graph construction. (A) de Bruijn graph where the nodes are k-mers and edges are k–1 overlaps between them. (B) Example of an Eulerian path that visits each edge (dotted arrows) exactly once in the graph (note: numbers represent the order of visiting edges). (C) Assembled reads corresponding to the edges that are traversed on the Eulerian path.

Assemblers following this paradigm start by extracting the set of all k-mers in the reads, which represents their k-spectrum. Each node represents a k-mer in the graph and each edge represents a k–1 overlap between the nodes. Ideally, when the traversal count of each edge is known, the Eulerian path that visits each edge in the graph exactly once corresponds to the entire chromosome (see Figure 4). Pevzner *et al.* proposed a slightly different representation of a de Bruijn graph where edges are corresponding to k-mers and nodes are corresponding to k-1 suffixes or prefixes of those k-mers [27]. While de Bruijn graphs can be constructed in a linear time algorithm, it is traversed in a polynomial time to find the optimal path on the graph [69].

This approach still needs improvements when assembling a genome with high-coverage and high-error profiles that increase the number of repeated and distinct k-mers respectively in the graph. Moreover, splitting reads into k-mers leads to the loss of information of k-mers contexts while it also increases the need for efficient storage and processing algorithms [34]. Finally, this graph is very sensitive to the k parameter [70]. The selection of the k-mer length should be sufficiently large to prevent false overlaps due to shared k-mers, but it should also be small enough to consider the true overlaps of shared k-mers. The k parameter should be selected according to the coverage of the reads and the average error profiles.

To overcome the need for the large hardware resources required to handle a graph of k-mers, various studies reformulate the representation of the de Bruijn graphs to ensure efficient storage in memory. Melsted *et al.* presented an approach for efficient memory usage based on the detection of a set of unique k-mers and storing them in a probabilistic data structure known as a Bloom filter [71]. Ye *et al.* introduced the idea of a sparse k-mer: rather than storing all k-mers in the memory, which is the case in de Bruijn graphs, a sparse subset of them is sufficient [34]. Conway *et al.* reformulated a de Bruijn graph as a bit map and represented each edge in the de Bruijn graph using one bit, which was set or cleared according to the existence of an edge [72]. While this representation has large memory requirements with large k values, a recent succinct representation of the de Bruijn graph that is independent from the k values has been proposed by Bowe *et al.* [73]. This representation is based on indexing and compressing graph nodes/edges using an extension of the Burrows-Wheeler transform.

This paradigm is implemented in several short-read assemblers such as Euler-SR [8]–[10], [26], [27], ALLPATHS-LG [7], [18], [59], Velvet [35], ABySS [31], SOAPdenovo [17], and SparseAssembler [34] (see Table 2, Table 3, Table 4).

### C. Greedy-Based Construction

**Figure 5:**
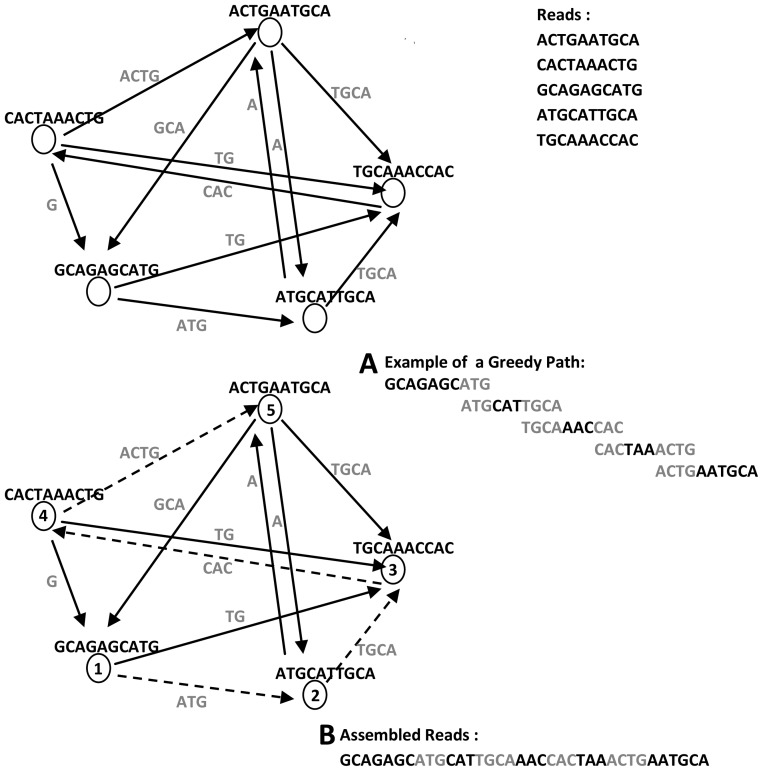
Greedy-based approach for graph construction. (A) Example of a greedy path (dotted arrows) that visits the nodes in the order of maximum overlap length (note: starting node is chosen randomly; at each node the greedy algorithm will choose the next visitor based on the maximum overlap length between this node and its connected neighbors). (B) Assembled reads corresponding to nodes that are traversed on the greedy path.

Greedy-based assemblers always make the choice with the greatest immediate contribution in solving sequence assembly problem. They follow the same basic operation: given any graph node, the assembler chooses the next visitor on its tour that maximizes the overlap length with the current node (see Figure 5). By using a set of heuristic techniques, greedy assemblers can detect false overlaps and high-scoring ones that are resulted from repetitive sequences. This approach is not widely used, since greedy assemblers do not consider any global information about read relationships and their paired-end links.

Greedy-based assemblers are suitable for small-size genomes. Using greedy approach for graph traversal may cause the algorithm to become stuck in local maxima, which produces a suboptimal solution for the assembly problem. The local maxima will increase the gaps between contigs in the assembly finishing process. A prefix tree is used to represent a greedy graph implicitly in some assemblers [33].

This paradigm is implemented in several short-read assemblers such as SSAKE [33], SHARCGS [12], VCAKE [74], and QSRA [75] (see Table 2, Table 3, Table 4).

### D. Hybrid-Based Construction

This approach has different perspectives, such as a hybrid between two different models of graph constructions that aims to increase the assembler's performance by exploiting the advantages of both models. A hybrid between OLC and greedy graph is implemented in Taipan [29] where nodes are the reads and edges represent the overlaps, and the graph is traversed to find a greedy path rather than a Hamiltonian path, as in the OLC approach [29], [44]. Greedy overlap–based assemblers use a greedy algorithm, which does not generally produce an optimal solution, but they achieve acceptable assembly quality as OLC assemblers using a moderate amount of hardware resources. Another perspective is combining different quality of reads from different sequencers in the process called hybrid assembly [28], [76]. Wang *et al.* proposed a pipeline for assembling reads from 454, SOLiD, and Illumina separately and combining their resulting contigs to build scaffolds and close gaps between them [32]. Cerdeira *et al.* proposed another pipeline for combining the contigs produced by different assemblers (i.e., Edena and Velvet) from different graph construction models such as OLC and de Bruijn to increase the assembly quality [77]. Moreover, the perspective of the hybrid approach between *de novo* and comparative assembly has been proposed for producing an efficient draft of assembled genomes [78].

## Graph Simplification Process

The graphs of high-throughput short reads contain huge numbers of nodes, edges, paths, and subgraphs. To overcome memory limitations and reduce computation time, the graph is simplified after the graph creation process [22]. Erroneous reads that are not recognized by the preprocessing filter form erroneous structures, which also complicate the graph and assembly process. These erroneous structures must be removed or simplified to prevent misassembled contigs and scaffolds.

**Figure 6:**
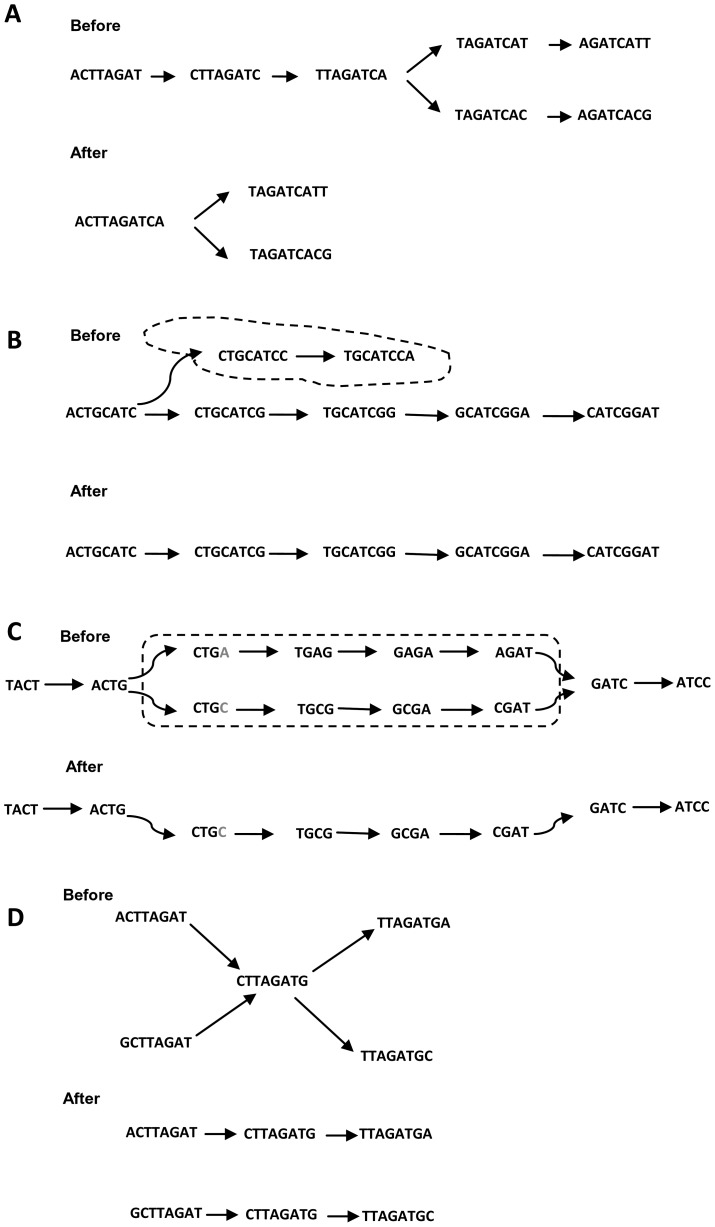
Different graph simplification operations. (A) Consecutive nodes are merged. (B) Dead end (dotted circle) is removed. (C) Bubble (dotted circle) is simplified where low-coverage path of the two paths that caused it was removed. (D) X-cut is simplified by splitting the connections into two parallel paths.

The graph simplification process begins by merging two consecutive nodes into one node, if the first node has one outgoing edge and the second node has one incoming edge (see Figure 6A). This simplification step corresponds to the concatenation of two character strings and it is similar to the approach taken by some overlap-based assemblers during graph construction [67].

Another simplification step involves the removal of the transitive edges [67] caused by oversampling of the sequencing technology. Given that there are two paths 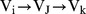 and 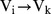, the path 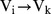 is transitive because it passes through 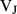 and it represents the same sequence as the first path, whereas the path 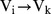 need not be represented in the graph because the path 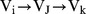 already exists in the graph. This is an important step in the graph simplification process, which reduces the graph complexity by a factor of the oversampling rate *c* calculated as 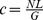, where *N* is the number of reads, *G* is the size of the genome being sequenced, and *L* is the length of reads [14], [29]. In the string graph, removing transitive edges is the step toward graph construction [13], [30], [60]. This simplification step is only applicable to the overlap-based graphs while the de Bruijn graph is naturally transitive-reduced.

Dead ends or spurs (tips) are different names for the same erroneous structures. The short dead-end paths are caused by low-depth coverage in the reads or the edges leading to the reads that contain sequencing errors and a mixture of correct and incorrect k-mers in the graph. To simplify this structure, some assemblers (e.g., Edena [14], ABySS [31], and CABOG [21]) test each branching node for all possible path extensions up to a specified minimum depth. If the path depth is less than a certain threshold, the nodes on the path are removed from the graph (see Figure 6B) [7], [8], [14], [17], [21], [35]. Other assemblers (e.g., SOAPdenovo [17], Velvet [35], and SGA [30]) remove the dead ends only if they are shorter than 2k and they have a lower coverage than other paths connected to a common destination node [17], [35], [79]. The value of k is sensitive to the removal of dead ends. Selecting a high value of k breaks the contigs in many places. Furthermore, it is difficult to determine the causes of dead-end branches, such as errors or a lack of k-mer coverage. If dead ends are caused by a lack of coverage, the process of removing them may lead to the removal of correct k-mers, which shortens the contigs.

Bubbles or bulges are caused by nonexact repetitions in genomic sequences or biological variations, such as SNPs (i.e., single base substitution). On the graph, their structure is a redundant path, which diverges and then converges. Fixing a bubble involves removing the nodes that comprise the less-covered side, which simplifies the redundant paths into a single one. The process of fixing bubbles begins by detecting the divergence points in the graph. For each point, all paths from it are detected by tracing the graph forward until a convergence point is reached. Finally, these paths are filtered according to their own k-mer coverage, quality scores, etc., or aligned with each other to determine their shared consensus bases. The paths with low coverage are removed from the graph and recorded in the log files for later use when extending contigs to scaffolds (see Figure 6C) [17], [35], [59]. While ABySS restricts the size of the bubble to n nodes (k≤n≤2k), SOAPdenovo [17] and Velvet [35] use a modified version of Dijkstra's algorithm to detect it. In addition, rather than reducing the bubble with redundant paths into a single simple path, some assemblers preserve the heterozygotes encoded in the bubble by using constrained paired-end libraries (e.g., ALLPATHS-LG [59]) or keeping the best two paths that are covered by the most sequencing reads (e.g., Fermi [60]).

X-cuts or tangles are formed in the regions of repeats, which allow more than one possible reconstruction of the target genome. The simplification of repeats is affected by their length because the length of any repeat can be between k and the read length. Tiny repeats with equal incoming and outgoing edges N, which are shorter than the read length, are resolved by removing the repeated nodes and splitting the connections into N parallel paths (see Figure 6D). The path partitioning is guided by mapping reads back to the edges (read threading) or mapping paired-end reads (mate threading). Euler-SR [10] and SOAPdenovo [17] resolve simple tangles using read threading technique. However, long repeats that exceed or equal the read length complicate the graph and produce multiple exponential paths between the nodes. Tracing all of these paths for finding the correct arrangement of reads is computationally expensive under the standard hardware resources. Based on the paired-end constraints, there is only one path that satisfies them between any nodes so the repeat may be resolved [8]–[10], [17]. Euler-SR [10] and ALLPATHS-LG [59] resolve more complex tangled repeats using mate threading technique, while Velvet integrates the Pebble and Rock Band algorithms to solve them using insert length distributions and mixing long and short reads, respectively [79].

Other graph simplification approaches targeted nonrecognizable erroneous structures such as erosion of erroneous edges formed by chimeric sequences [10], [35], deletion of sequences not covered by paired-end reads [59], and keeping only the edges that maximize the overlap length with other reads in the graph [14].

## Postprocessing Filtering

After finishing the graph simplification process, the graph is traversed to build longer reads known as contigs. Contigs are connected to form super-contigs or scaffolds. The process of building scaffolds is not easy. The graph is filtered and simplified to create correct contigs, which must be filtered and simplified before building the scaffolds [9]. The goals of postprocessing filtering are building contigs, filtering them, detecting misassembled ones, and correcting them to form scaffolds. Paired-end reads are used as a guide map to order and orient contigs during the scaffolding process. Appropriate contigs are joined together to form scaffolds depending on the positions of the paired-ends in the contigs, their orientation, and expected insert size. If two pairs are present in the same contig, their location and the distance between them must be matched using the information available in the paired-end libraries. If two pairs occur many times in contigs, the information about their orientation and insert size can be used to filter the choice of appropriate contigs to join them together. Paired-end data is also useful for detecting chimeric contigs where two or more regions from different genomic locations are misassembled into one contig. The frequency of paired-end links is also used as a filter criterion for removing misassembled contigs [52]–[55], [57], [80]. Contigs containing repeats can violate paired-end constraints and lead to misassembled scaffolds. Detecting these contigs early by tracing high-coverage regions that may reflect repeats in the contigs and removing them from the assembly set can prevent scaffolds from being misassembled.

**Figure 7:**
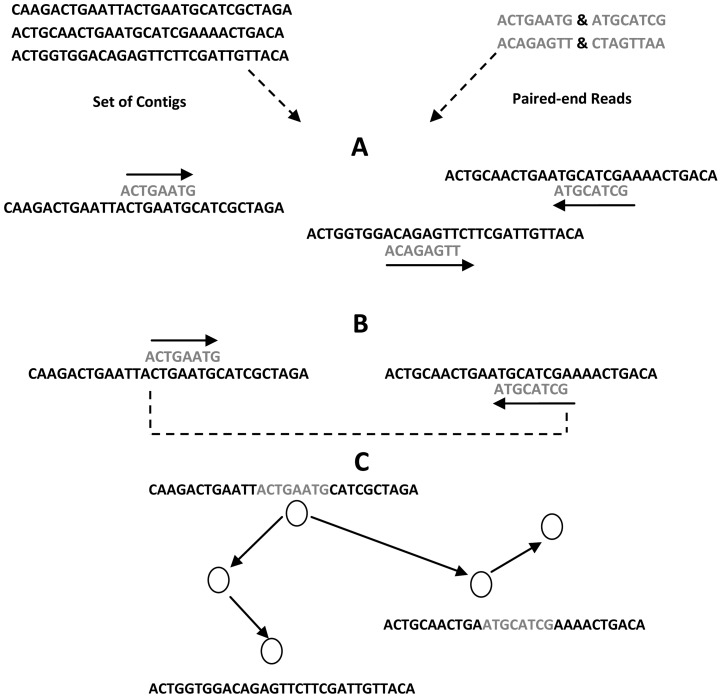
Building scaffolds using contig connectivity graph. (A) Paired-end reads are aligned to contigs and their orientations are determined. (B) The library insert size (dotted line) is determined between two pairs and compared with the one saved previously. (C) Contig connectivity graph is constructed and filtered according to paired-end constraints.

The goal of any scaffolding algorithm is to minimize the inconsistency between the assembled contigs and paired-end constraints based on majority voting from a large number of paired-end reads. Achieving this goal is NP-hard but there are useful heuristics for overcoming these challenges [21], [23], [42], [79], [81]. There are two approaches to building scaffolds. The first approach uses the graph built during the graph construction process (e.g., a de Bruijn graph) and integrates paired-end constraints to detect scaffold paths on the same graph [82]. Some assemblers align the paired-ends to contigs to detect those that can join together to form scaffolds [10]. Other assemblers use heuristic approaches to incorporate paired-end constraints into a de Bruijn graph [7], [35], [79], [83]. The second approach constructs a contig connectivity graph (also known as a scaffolding graph) (see Figure 7) where the nodes represent contigs and the edges encode paired-end constraints. This graph needs simplification and reduction because it contains cycles (redundant contigs), as well as transitive, associative, and erroneous edges (misassembled contigs) [15], [17], [30]. The scaffolding graph is usually traversed using a greedy approach, which visits the contigs in order to maximize the supporting paired-end constraints [80] or contig lengths [52].

If the gaps between the contigs are not filled with other contigs, they are filled with N characters that denote unknown bases between them and the total number of N can be estimated easily using paired-end constraints [53]. Some assemblers include scaffolding modules (e.g., Euler-SR, ALLPATHS-LG, Velvet, SGA, SOAPdenovo, and Shorty) (see Table 2) while others are stand-alone scaffolders such as Bambus [43], [56], SSPACE [52], SOPRA [53], MIP scaffolder [57], Opera [55], and SCARPA [54] (see Table 5).

**Table 5:** Postprocessing filters (“scaffolders”): Practical and technical comparisons.

Postprocessing Filters	Operating System	Programming Language	Single PC/Cluster	Open-Source	Paired-End Libraries	Input File Formats	Output File Formats	Websites	Ref.
Bumbus2* **(V2.0)**	Linux, Sun/Solaris Alpha/Ultrix Darwin OS X	C++, Python, Perl	Single	Y	Illumina, 454	AMOS***	fasta, agp, dot	http://amos.sf.net	[56]
SSPACE*	Linux	Perl	Single	Y (Basic v) N (Premium)	Illumina, 454, SOLiD	fasta/fastq	fasta	www.baseclear.com/bioinformatics-tools/	[52]
SOPRA **(V1.4.6)**	Linux (64) bits, Mac OS X	Perl	Single	Y	Illumina, SOLiD**	fasta, sam**	fasta**	http://www.physics.rutgers.edu/~anirvans/SOPRA/	[53]
Opera* **(V1.3.1)**	Linux	Java/C++	Single	Y	Any platform	fasta****, sam/bowtie	fasta	http://sourceforge.net/projects/operasf	[55]
MIP Scaffolder* **(V0.5)**	Linux (64) bits	C++, Perl	Single	Y	Illumina	fasta, sam	fasta	http://www.cs.helsinki.fi/u/lmsalmel/mip-scaffolder/	[57]
SCARPA*	Linux (64) bits	C++, Perl	Single	Y	Illumina, SOLiD	fasta, sam	fasta	http://compbio.cs.toronto.edu/hapsembler/scarpa.html	[54]

* Personal communications with authors.

** Users' experiences and communities' websites.

*** There are utilities available to import a variety of different data formats into AMOS.

**** For preprocessing, read files can be in fasta/fastq.

There are many challenges currently facing the stand-alone scaffolders such as using of different paired-end libraries with different insert sizes, dealing with different erroneous structures in the contig connectivity graph, which are resulted from sequencing errors in paired-end libraries, misassembled contigs and chimeric reads, resolving complex repeat structures, targeting metagenomic sequences, and utilizing efficient algorithms to solve the inconsistency among paired-end links. Further, similar to the error correction tools, there is a lack in the evaluation studies, which can assess different stand-alone scaffolders and compare them against built-in scaffolding modules using different paired-end libraries.

## Evaluating the Performance of Assemblers

Different assessment methods are used to evaluate the performance of existing assemblers from two perspectives. The first perspective is usability, which includes numerous issues such as hardware and software requirements, ease of installation and execution, user-friendly interfaces, and the speed of responsiveness to user commands [44], [84]–[86].

The runtime of an assembler and its memory usage are the most important issues for the usability measure. Depending on the available computational resources, current assemblers used in next-generation environments are classified into two categories. In the first category, the assemblers run on a single machine with very large memory requirements, e.g., to assemble human and mammalian genomes [17], [59]. In the other category, assemblers are run on tightly coupled cluster machines [31]. The high-throughput nature of next-generation sequencing technology due to short-read sequences and their quality scores imposes a major constraint on the system memory available. To ensure efficient memory savings, most assemblers formulate the assembly problem as a set of graph nodes and they rely on efficient data structures to accommodate these nodes. The different graph models were discussed earlier in the graph construction sections, particularly their advantages and disadvantages with respect to computational resources and several studies that reformulated their representations to ensure efficient storage in memory. However, no memory-efficient solution is available for NGS assemblers and there is a need for new tools and algorithms in this area.

**Figure 8:**
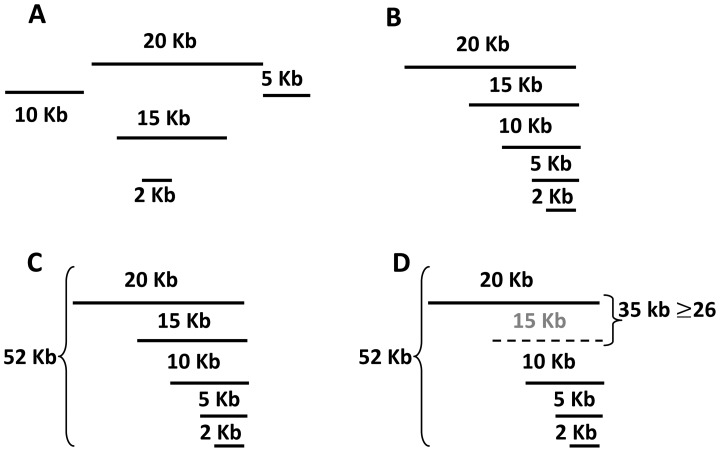
N_50_ calculation method. (A) Set of contigs with their length. (B) Contigs are sorted in descending order. (C) Lengths of all contigs are added (20+15+10+5+2 = 52 kb) and divided by 2 (52/2 = 26 kb). (D) Lengths are added again until the sum exceeds 26 kb, and hence exceeds 50% of the total length of all contigs: 20+15 = 35 kb≥26; then, N_50_ is the last added contig, which is 15 kb.

The second perspective is assembly quality, which mainly assesses the contiguity, consistency, and accuracy of the assembled genomes using different approaches. Several studies have measured the contiguity of assembled contigs and scaffolds using different statistical metrics to calculate their length distributions [87]–[94]. These metrics include the N_x_ score; the number of assembled contigs/scaffolds (a low number is usually preferred because it reflects greater connectivity); the maximum, minimum, and average lengths of the resulting contigs/scaffolds; the total short read lengths; and the sum of contigs/scaffolds. N_50_ (see Figure 8) is the most common statistical metric. A larger N_x_ score is usually better but it might not reflect the assembly quality because incorrect joints in the assembled contigs will increase the score [94].

Consistency measures aim to check the consistency between assembled genomes and the constraints imposed by paired-end libraries [95], read coverage [96], optical maps [97], or haplotype sequences [90]. They aim to assess the quality of the assembled genome by comparing it with a similar completed genome [89], [90] or by comparing its genetic data with independent genomic components from the same organism, such as mRNA or cloned genes, which are available in the public databases [87], [98]. If sequences are not available from the same organisms, the conserved sequences of related organisms may be used to determine the accuracy of the assembly and to detect conserved sequences in the newly assembled genome [87]. If a reference genome is available, the accuracy of the assembled genomes can be assessed by aligning the draft genome assemblies and reference genomes using different genomic alignment tools [14], [35], [44], [99]. The alignment process is useful for detecting different factors in the assembled genomes and it is used by some assessment metrics such as the percentage of reference coverage [17], [44]; the accuracy of contigs/scaffolds and their long-range contiguity [59]; the patterns of insertions, deletions, and substitutions [100]; and core and innovative genes [98].

Some evaluation studies have used a combination of previous methods to assess draft genome assemblies. Assemblathon [101] used previous metrics and defined its own new ones such as NG_50_, which is computed using the average lengths of haplotypes instead of the contig lengths used by N_50_; CPNG_50_/SPNG_50_, which is the average lengths of contigs/scaffolds that are consistent with haplotype sequences; and CC_50_, which is an indication of the correct contiguity between two points in assembled genomes. GAGE [63] used the E-size metric, which is the expected length of contig/scaffold that contains a randomly selected base from a reference genome. GAGE also reported that the evaluation process was affected by the quality of the datasets being assembled and the assembler/genome selected. Moreover, the statistical methods did not reflect the quality of the assembly process in terms of their accuracy and contiguity.

In addition to the previously discussed factors that affect the quality of the genome being assembled, other studies have used the sequencing coverage, the average length of reads, and the rate of sequencing errors in assessments [102]. They also used the scoring scheme to rate the different operations that reflect the accuracy of the assembled genome, such as insertions, redundancy, reordering, inversions, and relocations. There is usually a tradeoff between contiguity and accuracy, where maximizing one of them will impair another measure. Recently, a new metric, based on aligning paired-end reads to an assembled genome, had been proposed to generate Feature-Response Curves (FRC) to overcome this tradeoff [103], [104].

The choice of assembly algorithm and the complexity of the dataset being assembled will also affect the performance of an assembler. Different assemblers handle the errors and inconsistencies in datasets differently. These inconsistencies are caused by the variation between haploid and diploid genomes, and they depend on the frequency of heterozygosity. Thus, selecting the appropriate assembly algorithm and setting its parameter, such as k-mer size and minimum overlapping length, affects the quality of the genome assembly [25], [44], [105].

Zhang *et al.* [44] stated that de Bruijn graph–based assemblers are more suitable for large data sets, of which SOAPdenovo produces good assemblies for very short reads while ALLPATHS-LG is recommended for longer reads of 100 bp. In addition, greedy-based and OLC assemblers perform well for small data sets with very short reads and longer reads, respectively, under limited computational resources. Further, hybrid-based assembler Taipan delivers better results in terms of the assembly speed with the existence of sufficient memory. While SOAPdenovo has complicated configuration files, greedy-based assemblers and hybrid-based ones are superior in terms of easy software installation.

The recent version of Assemblathon competitions [106] reported some practical considerations for *de novo* assembly, which are that the assembly results must be taken several times using different assemblers with different parameter settings to determine their confidence, considering different metrics during assessment process, choosing the suitable assembler based on your interested metric (e.g., continuity, accuracy, coverage), evaluating the heterozygosity levels before starting your assembly run, and finally the contiguity metrics such as N_50_/NG_50_ or the assembly size may not be considered in the evaluation process, if you are targeting the genetic components in the assembled genomes.

Tables 1–5 offer a summary for different technical and practical issues such as the sequencing platforms, different input/output file formats, operating systems, programming languages, and open-source availability, which can help users and developers when choosing assemblers, error correction filters, or scaffolders.

## A Layered Architecture Approach for Building a General Assembler

After reviewing the four stages of the assembly process and a large number of NGS preprocessing filters, assemblers, and scaffolders, we identified the challenges of building a genome assembler from two perspectives: the user and the developer. For users, most current assemblers have command line interfaces that lack interactivity and user-friendly interface components. Furthermore, it is difficult to: write their commands correctly without syntax/semantic errors, prepare their input files in a format suitable for the assembler being used, or to adapt different parameter settings for different experiments because these are problematic tasks for nonexpert users. Moreover, users need assessment tools so they can assess the assembler's output and present their results in different formats with added statistical information, which are all issues related to the speed, accuracy, and efficiency of resource usage [44], [86]. Developers are struggling to increase the quality of assembled genomes and the usability of their assemblers with the computational resources available. They also need to address future improvements in sequencing technology and their new features, which means they have to develop innovative assembly strategies continuously, as well as efficient data structures [24], [84].

**Figure 9:**
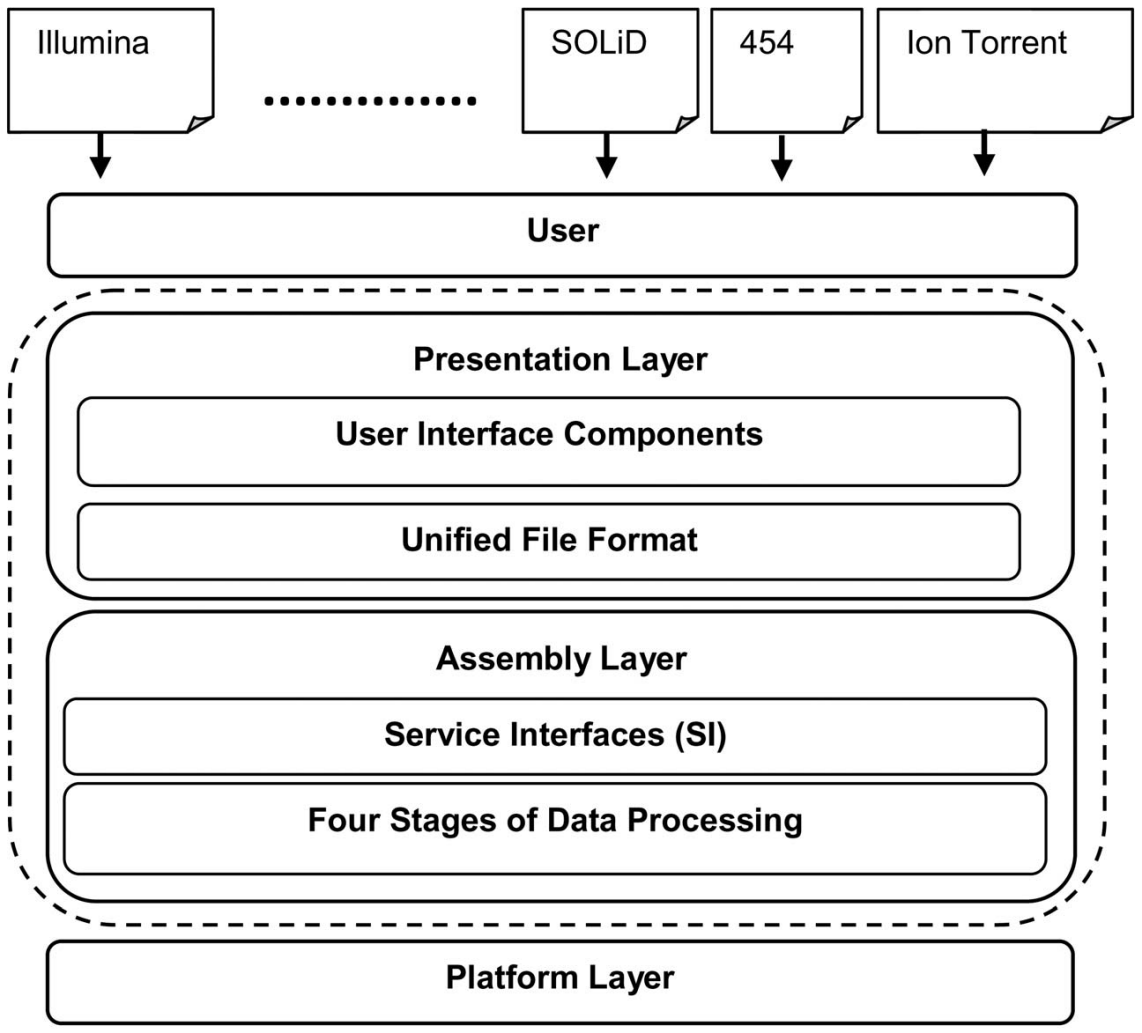
The proposed layered architecture for building a general assembler (dotted circle). This architecture has two basic layers: presentation and assembly layers. The presentation layer accepts the data from the user and outputs the assembly results through a set of user interface components. It is also responsible for converting platform-specific files to a unified file format for the underlying processing layers. The assembly layer contains three basic services: preprocessing filtering, assembly, and postprocessing filtering, which are provided through the four stages of the data processing layer. These services are supported through a set of communicated interfaces corresponding to each sequencing platform.

Based on these two perspectives, we suggest a layered architecture approach to building a general assembler (see Figure 9). A general assembler should be able to work with the wide range of NGS data generated by different NGS platforms and perform the four stages of NGS data processing. This architecture contains two basic layers, i.e., a presentation layer and an assembly layer, which contains different modules.

The presentation layer is responsible for taking the user inputs through a set of user interface components. It is also responsible for converting platform-specific files to a unified file format such as a fastq/fasta-like format, or a tool-defined format. This can be achieved by including an input module that deals with the data generated from each platform independently and exporting it in a unified format that can be processed in the subsequent layers in the same way, a feature that is already present in some tools [9].

The assembly layer covers the four stages of data processing, which we have discussed throughout our review, i.e., preprocessing filtering, graph construction, graph simplification, and postprocessing filtering. Many services are provided in this layer, e.g., preprocessing to correct error reads only, an assembly service to assemble reads and produce contigs, and postprocessing to build scaffolds. The implementation of these services relies on available approaches such as k-mers, overlap, sparse k-mers, and new ones. Each platform has different characteristics that affect the implementation of each service, such as the read length, error rate, error model, and sequencing depth (coverage), so a set of specified interfaces for each platform should be available and each service can implement multiple interfaces. These interfaces can also be used to deal with different types of sequences such as transcriptomes and metagenomes. Furthermore, it should be possible to exploit the complementary attributes of different sequencing platforms if necessary to integrate them into a hybrid assembly.

The modularity design [107] of the proposed general assembler makes it possible to use the existing implementations of the available services such as Bloom filter, FM-index, sparse k-mers, or defines new ones without affecting other modules in a flexible manner. In addition, it can be easily integrated with other models such as Trackster [108], via a set of communication messages through the presentation layer, to benefit from its visualization and analysis capabilities for assessing the values of different assembly parameters (e.g., overlap length, k-mer size), according to different characteristics of the employed data sets. Further, the general assembler can benefit from SAM/BAM [109], [110] file formats, which describe short-read sequence alignments in a text/binary format respectively. These formats are used with SAMtools to increase its usability across different built-in utilities for indexing, sorting, merging, etc. Moreover, the general assembler can utilize the standard format for genome assembly, fastg [111], which encodes different assembly graph notations such as nodes, edges, and paths and provides useful insights about different cleaning operations, different allelic variations, and assembly uncertainty. By supporting fastg through the unified file format layer, the general assembler can work directly on the graph structure produced from different assembly runs and perform hybrid assembly in an efficient manner. Since the target of this model is organizing the assembly process as a set of communicated layers with their supported services, the details of implementing the general assembler are left to the developers.

## Conclusions

Building an assembler for the next-generation environment presents many difficult challenges, such as the high-throughput nature of sequencers, short-read lengths, sequencing errors, and genomic repeats, which complicate the genome assembly task and increase the need for hardware resources. Furthermore, the settings of the assembly parameters differ according to the sequencing platform, error model, sequence reads, available resources, user definition, etc. Current assemblers still lack interactive user interfaces, easy setup requirements, and independence from the operating system, which are challenges for normal users with limited informatics backgrounds. Developers are struggling to develop innovative assembly strategies and efficient data structures to overcome the limitations of computational resources and the different types of NGS data generated by different sequencing platforms. In this review, we discussed next-generation genome assembly as a single coherent framework that comprises four basic stages: preprocessing filtering, a graph construction process, a graph simplification process, and postprocessing filtering. This approach to the assembly framework helps assembler designers to identify the basic challenges in each stage and to define their positions depending on their designs. This model can readily be extended to accommodate additional layers with new modules to handle metagenomic or transcriptomic sequences, or compressing some of its layers in a flexible manner can contract it. Furthermore, this four-stage framework can be used as the basis for building a general assembler for the NGS reads generated using different NGS platforms. The solution to the genome assembly problem begins by clearly identifying how these stages communicate with each other to deliver the final assembled genome. Therefore, building an assembler as a set of layers with clearly defined inputs, outputs, and communication messages will facilitate the development of innovative, interactive, and independent assemblers for the next-generation environment.
